# Effects of Two Fullerene Derivatives on Monocytes and Macrophages

**DOI:** 10.1155/2015/915130

**Published:** 2015-05-19

**Authors:** Sabrina Pacor, Alberto Grillo, Luka Đorđević, Sonia Zorzet, Marianna Lucafò, Tatiana Da Ros, Maurizio Prato, Gianni Sava

**Affiliations:** ^1^Department of Life Sciences, University of Trieste, Via L. Giorgieri 5, 34127 Trieste, Italy; ^2^Department of Chemical and Pharmaceutical Sciences, University of Trieste, Via L. Giorgieri 5, 34127 Trieste, Italy; ^3^Callerio Foundation, Institutes of Biological Research, Via A. Fleming 22-31, 34127 Trieste, Italy

## Abstract

Two fullerene derivatives (fullerenes **1** and **2**), bearing a hydrophilic chain on the pyrrolidinic nitrogen, were developed with the aim to deliver anticancer agents to solid tumors. These two compounds showed a significantly different behaviour on human neoplastic cell lines *in vitro* in respect to healthy leukocytes. In particular, the pyrrolidinium ring on the fullerene carbon cage brings to a more active compound. In the present work, we describe the effects of these fullerenes on primary cultures of human monocytes and macrophages, two kinds of immune cells representing the first line of defence in the immune response to foreign materials. These compounds are not recognized by circulating monocytes while they get into macrophages. The evaluation of the pronecrotic or proapoptotic effects, analysed by means of analysis of the purinergic receptor P2X7 activation and of ROS scavenging activity, has allowed us to show that fullerene **2**, but not its analogue fullerene **1**, displays toxicity, even though at concentrations higher than those shown to be active on neoplastic cells.

## 1. Introduction

Application of nanomaterials is increasing in the field of medicine with the aim to overcome the limitations of or to provide new tools and solutions to the existing approaches to human diseases [1, 2]. Among these nanoscale chemical structures, fullerenes represent an important source of the so-called biocompatible molecules because of their capacity to be in contact with cells and biological tissues without altering their behaviour [3]. Some of these substances were shown to be capable to cross cells without affecting their viability [4, 5]; others were demonstrated to be suitable as substrates for the growth of cells and tissues of importance for regenerative medicine and cell therapies [6]. They are also supposed to be good drug carriers in that they might use the enhanced permeability retention for selective accumulation of cytotoxic agents into solid tumour masses [7, 8].

**Figure 1:**
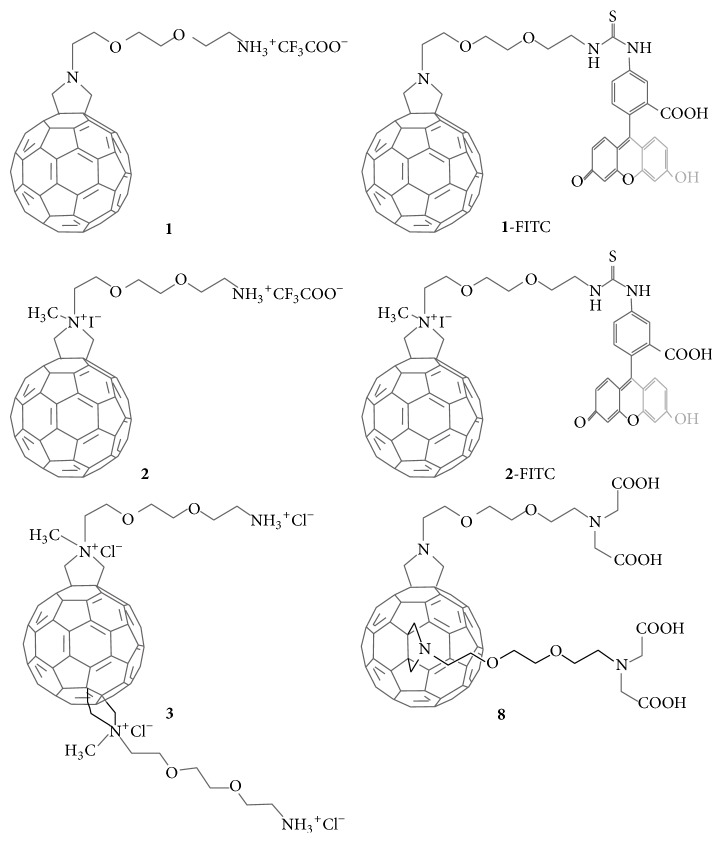
Chemical structures of the fullerene derivatives.

In this context, two fullerene derivatives (hereafter identified as fullerene** 1** and fullerene** 2**, Figure 1), bearing a hydrophilic chain on the pyrrolidinic nitrogen, were developed with the aim to deliver anticancer agents to solid tumours [9]. These two compounds showed a significantly different behaviour on cell cultures* in vitro*, as the charged compound** 2** is being significantly more cytotoxic than fullerene** 1**. A whole-transcriptome RNA-seq analysis, assessing their effects on gene expression in the human MCF7 cell line [10], highlighted the questions about the safety of fullerenes in biological systems. In fact, also those compounds (e.g., fullerene** 1**) which appear to be well tolerated, according to conventional functional studies, can cause important changes at the transcriptomic level, suggesting potential implications for the toxicity of these compounds. The effects of nanomaterials for immune cells have even major health, hazard identification and risk assessment implications. This is particularly important when proposing their possible use as drug delivery devices in tumor-bearing patients for whom the maintenance of an appropriate functionality of the immune system is of crucial importance for the benefit of the antitumor therapies. Then, independent of whether these fullerenes will be developed as drug delivery systems (e.g., compound** 1**) or for their antitumour properties (e.g., compound** 2**), the knowledge of their effects on cells of the immune system, also because these cells are involved in the recognition and scavenging of foreign material, appears crucial.

We therefore thought it is worth noting to study whether fullerenes** 1** and** 2** are biologically inert or they exert any biological effects on immune cells such as monocyte and macrophages. Depending on their surface modifications, fullerene derivatives may present quite different solubilities and, when put in biological systems, different proclivity to coalesce into sizes that could be readily recognized and captured by immune cells such as monocytes/macrophages with potential consequences on the biological functions of these cells. Monocytes and macrophages are cells known to be involved in both the innate and adaptative immune responses, the role of which is equally fundamental in the initiation and maintenance, likewise the resolution of many inflammatory processes. For this purpose, we tested circulating monocytes and cells resembling tissue resident macrophages, because they represent the first line of defence in the immune response to foreign materials, including fullerenes and nanostructures in general. Nanoparticles have been reported to be scavenged by macrophages before they transcytose across the plasma membranes of the target cells. Most of the study was then performed using monocytes and macrophages induced by differentiation of myeloid cell lines or primary cultures isolated from buffy coats.

## 2. Material and Methods

### 2.1. C_60_ Derivatives

The general synthesis of the fullerene derivatives is herein reported.

The C_60_ was functionalized using the 1,3-dipolar cycloaddition of azomethine ylides, generated by condensation of *α*-amino acid and aldehyde. In the case of derivatives** 1**,** 1**-FITC,** 2**,** 2**-FITC, and** 3**, the *α*-aminoacid prepared was the N-Boc-amino-diethoxy-ethylamino acetic acid, synthetized in 3 steps. Firstly, the 2,2′-(ethylenedioxy)bis(ethylamine) was monoprotected with di-*tert*-butyl dicarbonate. After purification, the product** 1 **was alkylated using benzyl 2-bromoacetate and, lastly, the amino ester was deprotected by catalytic hydrogenation to obtain the *α*-aminoacid with quantitative yield. The latter, together with paraformaldehyde, was used for the 1,3-dipolar cycloaddition on the fullerene C_60_. The Boc protecting group was cleaved using trifluoroacetic acid to obtain the free amino group with quantitative yield. For the synthesis of the fulleropyrrolidine derivatives** 2**,** 2**-FITC and** 3**, methylated on the nitrogen of the pyrrolidine ring, the methylation was done before the Boc deprotection to avoid the methylation on both nitrogen atoms. The introduction of the methyl group was done with methyl iodide, under heating in a pressure vial. The methylated derivative was then treated with trifluoroacetic acid to obtain the desired compounds. The fulleropyrrolidine was also coupled with fluorescein isothiocyanate isomer I (FITC). The positive charge on the deprotected amine was neutralized using diisopropylethylamine (DIPEA), making it available to attach the isothiocyanate group of the fluorophore compound. The product was precipitated from the reaction crude (DMF solution) with distilled MeOH subsequently washed with distilled MeOH. For further details, the full syntheses were already reported for** 1**,** 2**,** 3**, and** 8** [9] and for fluorescent derivative** 1**-FITC [11]. Compound** 2**-FITC was prepared following the same procedure performed for** 1**-FITC, using as starting materials compound** 2**.

Solubility of derivatives** 1** and** 2** has been measured in PBS and at acid pH. In the first case, the difference is not dramatic (3 *μ*M versus 4 *μ*M resp.). At pH 4, the solubility of** 1** increases by one order of magnitude (37 *μ*M) while derivative** 2** presented a solubility of 240 *μ*M.

### 2.2. Monocyte and Lymphocyte Cell Lines

The human monocytic U937 (cell line ATCC CRL 1593, Rockville, MD) and Burkitt's lymphoma BJAB cell line (kindly supplied by Dr. Macor, Department of Life Sciences, University of Trieste) were cultured in RPMI 1640 medium supplemented with 2 mM L-glutamine, 100 U/mL penicillin, 100 *μ*g/mL streptomycin, and 10% fetal bovine serum (FBS) (complete medium) and were subcultured three times a week for not more than 20 passages. Human monocytes and lymphocytes were isolated from buffy coats of different informed donors (in accordance with the ethical guidelines and approved from the ethical committee of the University of Trieste) as described by Bennett and Breit [12]. Briefly, the buffy coats were diluted 1 : 1 with PBS and added to an equal volume of histopaque-1077. After centrifugation for 30 min at 400 ×g without brake, the white band at the interphase between the plasma and the Histopaque fractions was soaked up, transferred into a sterile tube and washed twice with PBS. The cell pellet was then resuspended in RPMI_Hepes and transferred to a cell culture flask. The lymphocytes were recovered, as cells in suspension, after the monocytes were left to adhere for 1 h. The lymphocytes were used within two days, maintaining them in complete medium added with Hepes (25 mM), nEAA (1x), sodium pyruvate (1 mM), and 2ME (50 *μ*M). The cells were incubated in humidified air with 5% CO_2_ at 37°C.

### 2.3. Macrophage Induction

For induction of differentiation of U937 monocytes into macrophages (U937-PMA), the cells (1 × 10^6^ per mL) were seeded in the same medium and treated with 50 ng/mL of phorbol 12-myristate 13-acetate (PMA) at 37°C in an atmosphere of 5% CO_2_. After 72 h incubation, nonadherent cells were removed by aspiration. For test involving cell suspensions, the adherent cells (macrophages) were washed with PBS and then incubated at 37°C for 5 min in 5% CO_2_ with 0.05% trypsin and 0.02% EDTA·4Na solution to gently release them from the tissue culture flask. Recovered cells were then washed in PBS, resuspended in complete medium, and counted to prepare the cell suspension at the desired concentration. Differentiation of the human seeded monocytes from buffy coats into macrophages (MDM-LPS) was obtained following the same conditions described for U937; 10 ng/mL of* E. coli* LPS was used as differentiating agent for 5 days incubation in 5% CO_2_.

The characterization of LPS-induced macrophages was performed dosing IL12-IL10 (Platinum Elisa Human IL-12p70 “Ready-to-Use ELISA”; Human IL-10 Instant ELISA CE-IVD “Just add Sample”). In particular, we measured ≈100 pg/mL IL12 and ≈20 pg/mL IL10 that accordingly to Mosser '08 correspond to M1 polarization. The characterization was also confirmed by flow cytometry with human anti-IL-12 (p40/p70) and human anti-IL-10 antibodies (MACS, Miltenyi Biotec, Italy).

(All chemicals, unless specified, were purchased from Sigma-Aldrich, Italy).

### 2.4. Cytotoxicity Assays

#### 2.4.1. MTT Assay

The colorimetric 3-(4,5-dimethylthiazol-2-yl)-2,5-diphenyl tetrazolium bromide (MTT) assay was performed to assess the metabolic activity of cells plated into 96-well culture plates (10^5^ cell/well) and treated with 0.5–25 *μ*M of fullerene** 1** or fullerene** 2** in complete medium for 24–72 h. At the end of treatments, fullerenes containing medium were removed and replaced with fresh medium; for the cytotoxic assay, 20 *μ*L stock MTT (5 mg/mL) was added to each well, and cells were then incubated for 4 hr at 37°C. The converted MTT dye was solubilised with acidic isopropanol (0.04 N HCl in absolute isopropanol). Absorbance was measured at 540 nm and 630 nm using a microplate reader (Automated Microplate Reader EL311, BIO-TEK Instruments, Vermont, USA). All measurements were done in triplicate and each experiment was repeated at least three times.

### 2.5. Flow Cytometry Assays

All flow cytometry measurements were carried out on a Cytomics FC500 (Beckman Coulter Inc., Fullerton, CA), equipped with an argon laser (488 nm, 5 mV) and standard configuration with photomultiplier tube (PMT) fluorescence detector for green (525 nm, FL1), orange (575 nm, FL2), or red (610 nm, FL3) filtered light. After acquisition, of at least 10,000 events per each run, data are stored as list mode files and analyzed with the FCS Express V3 software or, the FL3 saved histograms, and were submitted to the analysis of the cell cycle, performed by the MultiCycle software.

#### 2.5.1. Apoptosis/Necrosis Assay

U937-PMA or monocytes/MDM-LPS cells were differentiated in 12-well tissue culture plates, as described above, and subsequently cells were incubated with the test compounds at 37°C in 5% CO_2_. At the end of treatment, cells were washed to remove extracellular fullerenes and stained with the appropriate probes described below to detect the cellular damage.

- DiOC_6_ (3,3′-dihexyloxacarbocyanine iodide) (FluoProbes, Interchim, Montlucon Cedex, France) was added (50 nM) to cell cultures in the dark at 37°C for 15 min, washed twice with 2 mL of PBS, and then stained at room temperature in the dark with 10 *μ*g/mL propidium iodide (PI) (Sigma) for 10 min. Double stained cells were then analyzed by flow cytometry.
- JC-1 cyanine iodide probe (5,5′,6,6′-tetrachloro-1,1′,3,3′-tetraethyl-benzimidazolcarbocyanine iodide; Molecular Probes Europe BV, Leiden, The Netherlands) was used as previously described [13]; briefly, the probe (2.5 *μ*g/mL) was added to cells in suspension (RPMI 1640 medium with 10% FBS) by gentle vortexing and, after incubation for 15 min at 37°C in 5% CO_2_ in the dark, the cells were washed twice with prewormed PBS (37°C), resuspended in PBS, and immediately analyzed by flow cytometer, acquiring FL2/FL1 signals on viable cells (those excluding PI, run in parallel). JC-1 monomer was measured at FL1-PMT, JC-1 aggregated at FL2-PMT. Cells treated with 50 *μ*M of the uncoupler carbonyl cyanide 3-chlorophenylhydrazone (CCCP) at 37°C for 15 min were run in parallel as a control for the collapse of mitochondrial transmembrane potential.
- YOPRO-1 (Molecular Probes, Invitrogen) (2 *μ*M) was added to cell cultures in the dark at 37°C for 30 min. Recovered cells were washed twice with cold PBS, stained with PI, and read by flow cytometry. 3 mM ATP (adenosine 5′-triphosphate disodium salt) was used as agonist of the purinergic receptor P2X7R.

#### 2.5.2. Cell Cycle Analysis

0.5 × 10^6^ cells were fixed in 70% ethanol, washed twice with PBS, and allowed to balance in PBS for 1 h. Cells were stained overnight with 0.5 mL of a PBS solution containing 10 *μ*g PI, 0.25 ng FITC, and 4 *μ*g RNase (all chemicals were purchased from Sigma-Aldrich, Italy).

### 2.6. Cell-Uptake Assays

#### 2.6.1. Flow Cytometric Evaluation

Monocytes/MDM-LPS (10^6^/mL cells per well) were incubated in complete medium 24 h with 5–10 *μ*M** 1**-FITC or** 2**-FITC. The cells were then washed with PBS to remove the noninternalized compound and analysed by flow cytometry. For kinetic studies, cell suspensions (10^6^/mL cells per tube) in PBS were kept in thermostated bath and added with the** 1**-FITC; for each time point (0–60 min) of treated cells, appropriate controls were run in parallel and read by flow cytometry. A further series of tubes were incubated for 30 and 60 min, washed to remove unbound fullerenes, and subsequently incubated for 10 min with 1 mg/mL of the extracellular quencher Trypan Blue prior to flow cytometry measurement.

#### 2.6.2. Confocal Microscopy

Mononuclear cells were primed with LPS on coverslips (1 × 10^6^ cells per coverslip), placed in a 12-well plate and treated for 24 hr with 10 *μ*M** 1**-FITC or** 2**-FITC. Monocytes/MDM-LPS cells, adhered on coverslips, were then washed with PBS and the intracellular localization of fullerenes was traced using the mitochondrial marker Mito-ID red mitochondria (Enzo Life Sciences, EU) following the instructions of the manufacturer. Cells were examined using a Nikon C1-SI confocal microscope (TE-2000U) equipped with a 60x oil immersion lens.

### 2.7. ROS Production

After treatment, with 10 *μ*M of the test fullerenes, the recovered cells were washed and concentrated (20 × 10^6^/mL in PBS) prior to staining with 10 *μ*M CM-H2DCF-DA probe (Molecular Probes, Invitrogen, Italy), for 30 min, at room temperature and in the dark. At the end of the incubation time, cells were washed twice and diluted to 10^6^/mL in RPMI-1640 modified without phenol red and each sample (treated or control cells) has been divided into 2 aliquots (0.5 × 10^6^ cells/0.5 mL), one representing the basal production of ROS and the other challenged with PMA at *T* = 0. From both groups, a kinetic analysis by flow cytometry was run from 0 to 60 min for the ROS production.

### 2.8. Statistical Analysis

Data obtained from repeated experiments were subjected to computer-assisted analysis using GraphPad InStat 3, and statistical significance was assumed at *P* ≤ 0.05 (ANOVA, Student-Newman-Keuls posttest). For cytotoxic assays, IC_50_ values were extrapolated by regression correlation analysis performed by GraphPad InStat 3 from experimental curves concentration effect (*r* ^2^ ≥ 0.9).

## 3. Results and Discussion

### 3.1. Cytotoxicity and Cell Cycle Effects on Primary Cells and on Lines of Lymphoid and Myeloid Derivation

The cytotoxicity (measured by the MTT) of the test compounds (Figure 1) was carried out on fresh peripheral blood monocytes and lymphocytes in comparison to macrophages and to cell lines of lymphoid and myeloid origin. The IC_50_ values reported in Table 1 show a very low susceptibility of primary cell cultures, composed of resting cells and macrophages from buffy coats to the effects of fullerenes** 1** and** 2**. These cells showed no toxicity up to 72 h exposure to the maximum dose tested, and it was not possible to precisely extrapolate the IC_50_ in these cell lines. Concentrations higher than those tested could not be used because of the formation of aggregates when the solutions of** 1** and** 2** were put in contact with the cells. On the contrary, fullerenes** 1** and** 2** show a measurable cytotoxicity for the stabilised cell lines, including the PMA differentiated U937 cells (U937-PMA), slightly superior for fullerene** 2** in the U937 and BJAB cell lines, similar to previous studies with MCF/7 cells [9]. When cell exposure was extended to 72 h, 25 *μ*M fullerene** 2** showed even greater cytotoxicity, corresponding to about 75% of cell loss (O.D. fullerene** 2**: 0.197 ± 0.016 versus O.D. controls: 0.791 ± 0.02, *P* < 0.001). The effects on these proliferating cells confirm the highest toxicity for fullerene** 2** and are already evident at concentrations below the IC_50_. The cell cycle analysis of U937 cells exposed to 10 *μ*M fullerenes** 1** and** 2** (approximately 50% of their IC_50_ in this cell line) showed the slow-down of cell progression into the cell cycle phases at any point of examination and without a particular phase specificity; compound** 2** was much more active than** 1** also in this experiment (Table 2).

The MTT test has been reported to present some limitations to accurately predict fullerene toxicity. It works poorly with C_60_ itself and better with C_60_ derivatives [14]. It has then been suggested that more than one assay might be required when determining nanoparticle toxicity for risk assessment [15]. Our study was then extended to the effects of fullerenes on monocytes and on macrophages using further methods suitable to define more precisely their activity on cell viability. These tests were done on the resting population of monocyte-macrophages obtained from buffy coats in order to study the potential cytotoxic effects of the test fullerenes on cells that can be found* in vivo* and which have a role in the pharmacokinetics of nanomaterials.

### 3.2. Effects on Mitochondria and on Cell Membrane

The JC-1 cyanine dye is a suitable probe to measure the fall of mitochondria energy in response to cytotoxic drugs. Flow cytometry measurements of the ΔΨ*m* (mitochondrial membrane potential), with two different colours (green/red), allows us to distinguish the formation of the JC-1_aggregates (given by the FL2) and of the JC-1_monomer (FL1) formed in the mitochondria of the treated cells. Compounds** 1** and** 2** were analysed on primary cultures of resting monocytes and on macrophages resembling the M1 polarized (MDM-LPS) macrophages.

**Figure 2:**
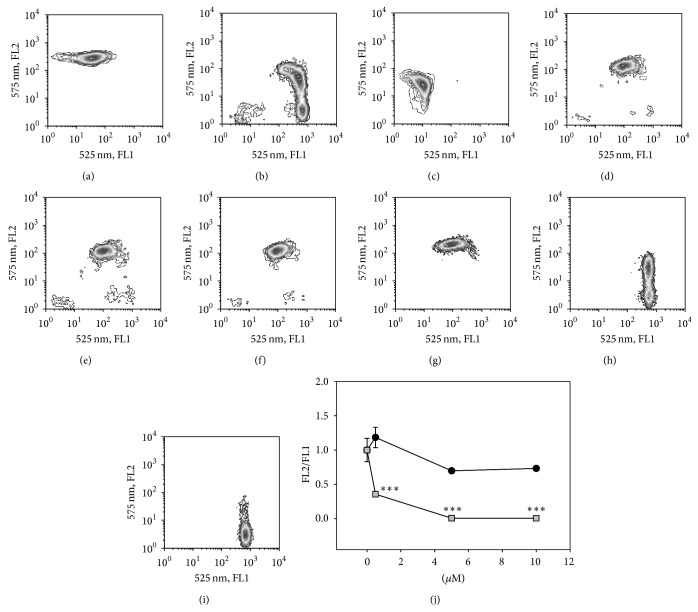
Analysis of the energized mitochondria of MDM-LPS and monocytes stained with the cyanine dye JC-1. Dot plots show JC-1_monomer FL1 (*x*-axes) and JC-1_aggregates FL2 (*y*-axes) of untreated ((a) MDM-LPS, (c) monocytes) and positive ((b) CCCP) controls; fullerene** 1 **treated MDM-LPS at 0.5 *μ*M (d), 5 *μ*M (e), and 10 *μ*M (f). Fullerene** 2 **treated MDM-LPS, respectively, at 0.5 *μ*M (g), 5 *μ*M (h), and 10 *μ*M (i). (j) displays the ratiometric assessment of mitochondrial polarization signals as FL2/FL1 (JC-1_aggregated/JC_1 monomer) of MDM-LPS cells treated for 24 hrs with fullerene** 1** (circle) and fullerene** 2** (square) at the concentrations shown on *x*-axes. Mean values ± SEM of at least three independent determinations: ^*∗∗∗*^ *P* < 0.01 versus untreated controls, post-ANOVA Student-Newman-Keuls multiple comparison test.

Monocytes and MDM-LPS were treated for 24 h with 0.5–10 *μ*M fullerenes and subsequently stained with the metachromatic probe. CCCP was used as positive control (Figure 2(b)). Derivative** 1** does not significantly modify the treated cell population and the cytograms of the treated cells (Figures 2(d)–2(f)) are comparable to those of the untreated controls (Figure 2(a)). Conversely, 5 *μ*M fullerene** 2** (Figure 2(h)) causes the mitochondrial depolarization in approximately 50% of the treated cells, as evidenced by the loss of aggregated JC-1. The use of 10 *μ*M leads to a complete effect in 100% of the cell population (Figure 2(i)). If we consider the JC-1_aggregated/JC_1 monomer ratio (FL2/FL1) (Figure 2(j)), the ratiometric semiquantitative assessment of mitochondrial polarization state caused by fullerene** 2** shows a statistically significant drop of the transmembrane energy potential of the mitochondria in comparison to the untreated control with any dose tested. The two fullerenes, tested on monocytes, characterised by a lower energetic status (Figure 2(c)), show no effect on these cells (data not shown).

**Figure 3:**
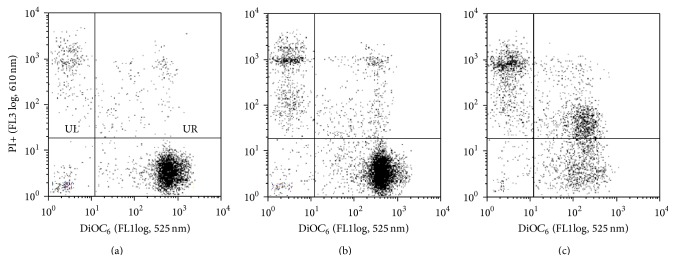
Analysis of the apoptosis/necrosis by DiOC_6_/PI double staining. Representative dot plots showing MDM-LPS cells untreated (control, (a)) or after 24 h treatment with 25 *μ*M fullerene** 1** (b) and fullerene** 2** (c). Quadrants: LR (DiOC_6_+/PI−); LL (DiOC_6_−/PI−); UR (DiOC_6_+/PI+); UL (DiOC_6_−/PI+).

**Figure 4:**
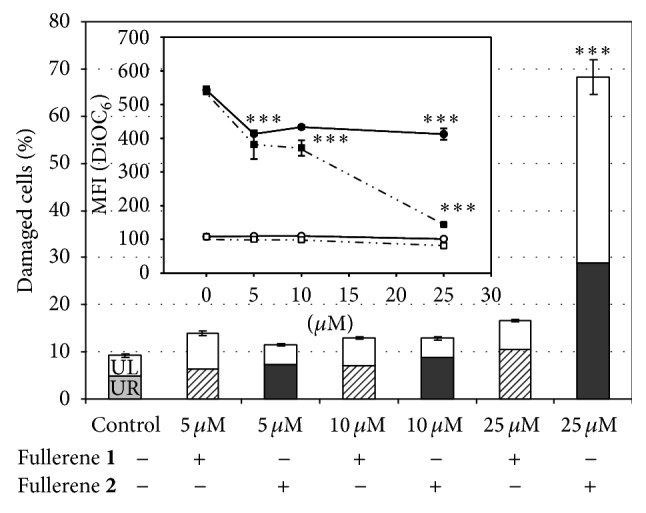
Effects of fullerene treatment on monocytes and MDM_LPS analysed by DiOC_6_/PI double staining. The figure shows the percentage of damaged cells measured in quadrants UR and UL (see Figure 3) after fullerene treatments while in the inset there are reports of the MFI values of MDM-LPS (filled symbols) or monocytes (open symbols) after treatment with fullerene** 1** (circle) and fullerene** 2** (square). Mean values ± SEM of at least three independent determinations: ^*∗∗*^ *P* < 0.05, ^*∗∗∗*^ *P* < 0.01 versus untreated controls, post-ANOVA Student-Newman-Keuls multiple comparison test.

The consistency of the depolarization induced by** 2** and the consequences of this effect for cell viability were analysed with the DiOC_6_/PI dual staining. This mitochondrial potential-sensitive dye renders the viable cells fluorescent (see Figure 3(a), untreated controls), because of their high transmembrane potential (lower-right quadrant), while its uptake is reduced in early and late apoptotic cells. The dual staining, DiOC_6_ combined with PI, allows us to distinguish cells in early stages of apoptosis (negative for PI with decreased DiOC_6_ fluorescence) from those in late stage of apoptosis (DiOC_6_+/PI+, Figure 2, upper-right quadrant, UR) and finally the necrotic cells that will be PI positive only (Figure 2, upper-left quadrant, UL). A representative experiment with fullerenes** 1** and** 2** is given in Figure 3 ((b): fullerene** 1** and (c): fullerene** 2**) and the detailed effects are reported in Figure 4.

The analysis of the effect of 5–25 *μ*M fullerenes** 1** and** 2** for 24, reported in Figure 4, confirms the absence of activity on monocytes (empty symbols) and the profound depolarization caused on MDM-LPS cells (filled symbols) (MFI values, indicative of the mitochondrial membrane polarization, insert of Figure 4). However, only a limited percentage of late apoptotic (UR) and necrotic cells (UL) are detectable up to 25 *μ*M of fullerene** 1** (Figure 4) whereas** 2** confirms its cytotoxicity with over 70% cells irreversibly damaged at 25 *μ*M concentration.

From these data, it can be concluded that the depolarization measured by JC-1 and DiOC_6_ probes, after exposure of cells up to 10 *μ*M, does not lead to cell death and that higher doses of the cytotoxic derivative** 2** are required to bring cells to complete their apoptotic pathway. Despite the hydrophobic nature of C_60_, that should allow the insertion of the tested compounds into the cell membrane bilayer, leading to potential alteration of its structure and function, there is no increase of permeability to PI, suggesting that the observed cell toxicity largely depends on the effects on mitochondria. In fact, we showed fullerene** 2** to inhibit the “mitochondrial target of rapamycin” (mTOR) pathway in MCF7 cells, an important intracellular signalling cascade regulating cellular metabolism, growth, and proliferation in response to the cellular energetic and oxygen levels and to a number of other stimuli [10].

The YO-PRO-1 probe was used to functionally detect the apoptotic macrophages, that become permeable to the fluorescent probe, but not to PI. YO-PRO-1 selectively enters throughout the P2X7R, an ion channel receptor that is activated by extracellular ATP and indirectly by a broad range of stimuli (bacterial and particulate material). ATP-gated P2X7Rs leads to a rapid caspase-1 activation.

**Figure 5:**
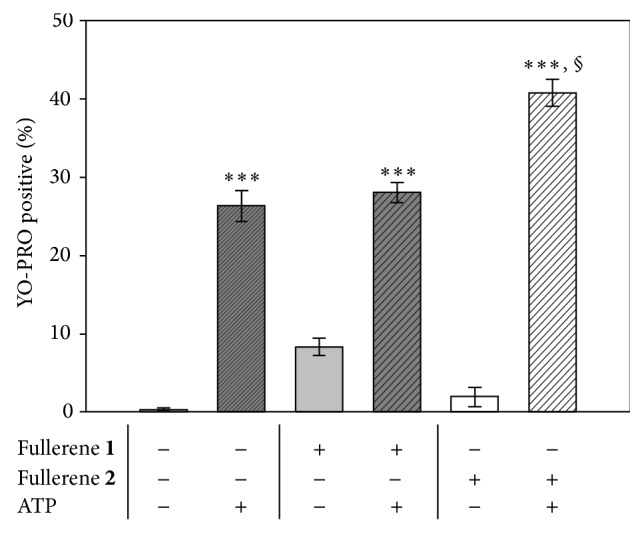
Evaluation of the P2X7R opening in MDM-LPS. Flow cytometric measurement of control and treated cells, with 10 *μ*M fullerenes (24 hr), stained with the YO-PRO fluorescent probe (solid histograms) or after addition of the agonist ATP (3 mM) (line pattern histograms). PI positive cells were gated out from the analysis and were below 10%. Mean values ± SEM of at least three independent determinations: ^*∗∗∗*^ *P* < 0.01 versus untreated/unstimulated controls (−) and ^§^ *P* < 0.05 versus ATP alone, post-ANOVA Student-Newman-Keuls multiple comparisons test.

MDM-LPS cells express functional P2X7R since they respond to exogenously added ATP (Figure 5, line filled histograms). By contrast, 24 h treatment with 10 *μ*M fullerenes** 1** and** 2** did not induce the opening of the receptor which is in line with the absence of significant increases of apoptotic cells in these experimental conditions (see Figure 4). The addition of ATP to the MDM-LPS cells, pretreated with fullerene** 2**, significantly increased the percentage of YO-PRO-1 positive cells as compared to the use of ATP alone (Figure 5). During pathophysiological conditions, the endogenous release of ATP from necrotic cells, as extracellular ATP in the milieu (eATP), represents a danger signal that alerts and activates the innate immune response against the tissue damage. eATP binds the purinergic receptor P2X7 triggering the formation of a pannexin-1 hemichannel, resulting in the activation of the NLRP3 inflammasome [16], as reported for environmental irritants, including silica and asbestos [17, 18]. It could be argued that the pretreatment with** 2**, but not with fulleropyrrolidine** 1**, contributes to rendering the cells more responsive to ATP, leading to the pyroptosis [19] of MDM-LPS cells.

### 3.3. Binding and Internalization of Compounds **1** and **2**

**Figure 6:**
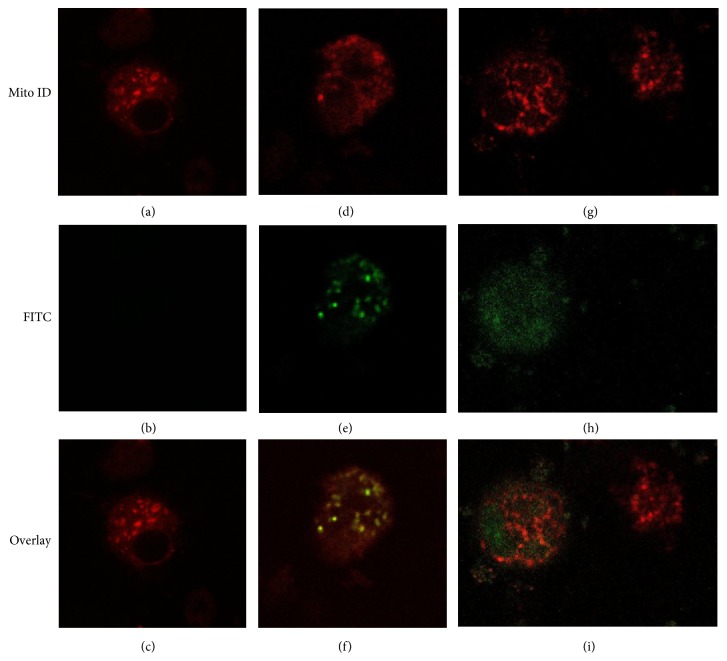
Confocal microscopy of MDM-LPS cells after 24 hr incubation with 10 *μ*M fullerene** 1**-FITC ((d), (e), and (f)) or fullerene** 2**-FITC ((g), (h), and (i)) or untreated control ((a), (b), and (c)), stained with Mito-ID (red fluorescence). Many fields were examined and over 95% of the cells displayed the patterns of the respective representative cells shown here.

Confocal laser scanning microscopy was used to study the internalization of 5 *μ*M and 10 *μ*M of the fluorescent derivatives fullerene** 1**-FITC and fullerene** 2**-FITC into human monocytes and MDM-LPS cells (Figure 6). After incubation for 24 h, the cell distribution of these two fullerenes showed compound** 1** mainly in the form of aggregates in the cytoplasm (Figure 6(e)) whereas compound** 2** was diffusely present in the cell cytoplasm (Figure 6(h)). The absence of colocalization with the MitoTracker probe suggests the absence of a direct localization of these fullerenes in the mitochondria. Also, the persistence of the fluorescence signal of FITC suggests that these compounds are not sequestered by lysosomes, into which the acidic environment, within 24 h, would have caused the FITC degradation.

Uptake studies, carried on by flow cytometry analyses (Table 3), confirm the selective and concentration-dependent binding of the tested compounds to macrophages (>90%) than to monocytes. The high MFI values of fullerene** 1** treated cells should be attributed to the strong fluorescent signal (relative fluorescence unit, RFU) emitted by fullerene** 1**-FITC, greater than that of fullerene** 2**-FITC, as determined using equimolar solutions of the FITC-labelled fullerenes in an* in vitro* cell-free system (RFU fullerene** 1**/RFU fullerene** 2** = 4.1 ± 0.2).

**Figure 7:**
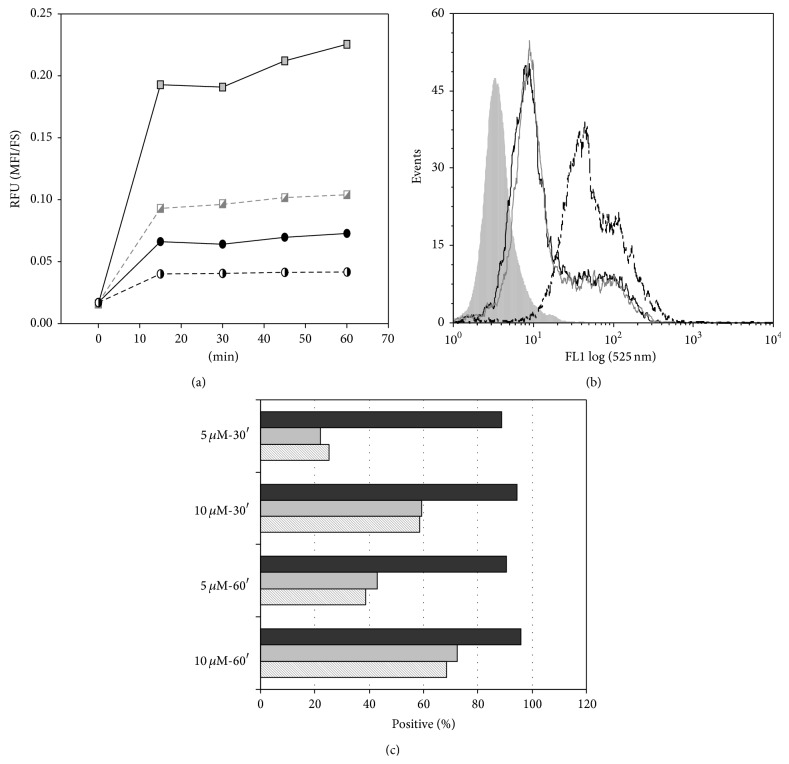
Flow cytometric analysis of the binding/uptake of fullerene** 1-**FITC with MDM-LPS and monocytes. In (a), the kinetic assay, carried out at 37°C for MDM-LPS (square) and monocytes (circle) treated with 5 *μ*M (half-filled symbols) or 10 *μ*M (filled symbols), is shown as MFI signal referred to the forward scatter parameter. Overlays of (b) are representative of untreated (gray filled) or fullerene** 1**-FITC treated cells (for 60 minutes with 10 *μ*M) without washout of unbound fullerene (black solid line) or after cell washing and addition of the TB quencher (gray and black thin lines). Histograms of (c) represent the percentage of positive cells stained with fullerene** 1**-FITC at the concentrations and times reported on *y*-axis without washing out of the fullerene (black), or after washing (gray) and subsequently addition of TB (line pattern histograms).

The kinetic study of** 1**-FITC (more efficient as fluorescent “tracer” and less toxic than** 2**-FITC) interaction with primary cultures of monocytes and MDM-LPS cells was done at 15 min intervals (Figure 7). The MFI values (Figure 7(a)) confirmed the specificity of fullerene** 1** binding to macrophages than to monocytes, even after normalization of the data on the cell dimensions (FS channel from flow cytometry data) given that macrophages are generally larger than monocytes. Compound** 1** binds to MDM-LPS in a concentration dependent way, reaching the plateau within 15 minutes of incubation. The entry of fullerene** 1**-FITC into the treated cells was determined after 30 and 60 min incubations, with the cells thoroughly washed to remove any remaining surface-bound fullerene and fluorescence and before and after the addition of the quencher Trypan Blue (TB) (Figure 7(b))** 1-**FITC interacted approximately with >90% of MDM-LPS already after short time exposure and independent of the concentration tested (Figure 7(c), black histograms). Theinteraction with MDM-LPS cells is rather weak and the positive cells are markedly reduced by simple washing. Washing is responsible for the loss of about 80% of fluorescence (MFI: 5 ± 0.4 versus 22.6 ± 0.7 for washed versus unwashed, resp., at 5 *μ*M) with 5 *μ*M** 1**-FITC. However, the fullerene measured after washing is inside the treated cells since the signal is not disturbed by the addition of the quencher TB (Figure 7(c), line filled histograms).

### 3.4. ROS Scavenging Activity of Fullerenes **1** and **2**

**Figure 8:**
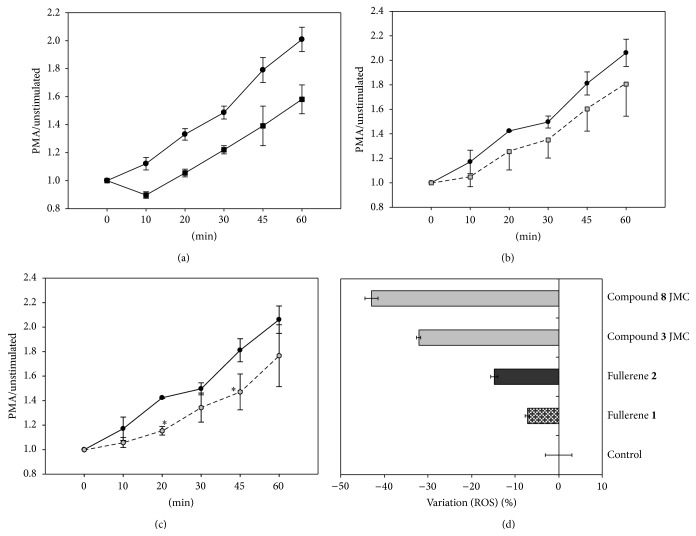
Evaluations of ROS produced by PMA-activated macrophages with the CM-H2DCF-DA probe. MDM-LPS response of controls to the NADPH activator PMA is shown in (a). The kinetic ROS production of fullerene** 1** (b) and fullerene** 2** (c) pretreated cells (gray symbols) is compared to that of untreated control (black symbols), and all (untreated and treated) were stimulated with 0.1 *μ*M PMA. Each point represents the mean value ± SEM of at least three independent determinations, obtained by the ratio of the fluorescence emitted by PMA stimulated cells/basal fluorescence, measured as MFI at each time of *x*-axes. ^*∗*^ *P* < 0.05* versus* untreated controls, post-ANOVA Student-Newman-Keuls multiple comparison test.

MDM-LPS cells can be compared to classically activated M1 macrophages [20] and, as expected, they respond to the PMA induced ROS production [21, 22], measured by the use of DC-H2DCF-DA, a probe becoming fluorescent in the presence of oxidants in viable cells (Figure 8(a)). MDM-LPS cells stimulation by PMA tended to reduce the ROS production, particularly when pretreated with** 2**, with a statistically significant inhibition of about 30–40%. This result is in agreement with the electron affinity properties of fullerenes C_60_, supporting their capacity to act as radical scavengers [23], depending on the functionalization that can change the photophysical electrochemical properties and their ROS-generating/quenching capacity [24]. Therefore, derivative** 2**, endowed with a solubility higher than** 1**, displayed a better quenching capacity. The lowest scavenging activity of** 1** might be further ascribed to its property to form nanoscale aggregates (that we observed at the confocal microscopy; see Figure 6) with reduced surface-to-volume ratio that could affect its ROS-quenching capacity [25]. Since covalently attached groups to C_60_ may play an important role in the ROS scavenging properties, the activity of fullerenes** 1** and** 2** was compared to that of two bis-functionalized derivatives, namely, compound** 3** and compound** 8** (see Figure 1). These bis-adduct compounds showed a significantly higher scavenging activity than** 1** and** 2** which is in line with the hypothesis relating the quenching capacity with the increase of C_60_ functionalization [24, 26].

## 4. Conclusions

The results of the present study stress the interaction of fullerene derivatives** 1** and** 2** with cells of the immune system endowed with important roles in the control of inflammatory and cancer diseases. The tested compounds are generally more active on neoplastic proliferating cells than on circulating monocytes, even though they get into macrophages.

It cannot be excluded that the interactions with these cells, even though at concentrations higher than those cytotoxic on tumour cells, may alter the capacity of macrophages to appropriately respond during the inflammation processes or to actively contribute to eradicate tumour cells. On the other side, this effect might be appropriately exploited to promote the pyroptosis with the aim to contain the tissue damage caused by an excessive maintenance of macrophage proinflammatory activity.

Among the compounds tested, fullerene** 2** is more toxic than fullerene** 1** for these cells. This cytotoxicity cannot simply be attributed to its insertion onto the structure of the membrane bilayer, as expected for this type of nanostructure. Compound** 2 **gets into the cells and can be found into the cell cytoplasm without altering the cell membrane permeability, even at doses active on tumour cells. Rather, the mechanisms by which it is toxic to macrophages must be attributed to the high concentration to which these cells were exposed that negatively interfered with the function of mitochondria. Alternatively, and at lower concentrations, cell toxicity might be viewed in the context of damaged tissue by increasing the eATP activated P2X7R pathway that finally leads to cell death by apoptosis.

For compound** 2**, the presence of a pyrrolidinium ring on the fullerene carbon cage brought to a more active derivative unlike the corresponding fulleropyrrolidine. In fact, compound** 1** interfered very weakly with the macrophage, once inside the cells, confirming that this slightly different functionalization plays a fundamental role for the biological activity of these compounds. These variations could be attributed to a better solubility of derivative** 2** and to a consequent lower tendency to form aggregates. Moreover, the introduction of a positive charge in close proximity of the carbon cage is known to enhance the electron-acceptor character of the fullerenes and this characteristic can lead to a better radical scavenger effect.

The fact that even minimal chemical differences between fullerene** 1** and** 2** may be responsible for a significant different toxicity of these nanostructures, often defined as biocompatible and easy to handle with biological tissues, should be taken into due consideration. In conclusion, compound** 1** showed biological properties that make it compatible with monocytes and macrophages, suggesting its potential use to deliver substances capable to modulate the immune responses or, as already proposed, as a vehicle to deliver anticancer drugs.

## Figures and Tables

**Table 1:** Fullerene cytotoxicity on primary and stabilized cells of lymphoid and myeloid derivation.

	Cell lines			Primary cultures		
	U937	U937_PMA	BJAB	Monocytes	MDM_LPS	Lymphocytes
Fullerene **1**	27 *μ*M	34 *μ*M	40 *μ*M	>>50 *μ*M	>50 *μ*M	>50 *μ*M
Fullerene **2**	17 *μ*M	32 *μ*M	24 *μ*M	>>50 *μ*M	>50 *μ*M	>50 *μ*M

Cells (10^5^/well) were treated in complete medium with fullerene **1** and fullerene **2 **for 24 hr and then subjected to MTT test. IC_50 _values were obtained (interpolation with GraphPad InStat) from data of repeated experiments; each of them are being with samples at least in triplicate.

**Table 2:** Cell cycle analysis of fullerene derivatives U937 treated cells.

	% G1	% S	% G2M
24 h			
Control	36.3 ± 0.7	59.9 ± 0.9	3.9 ± 0.2
Fullerene **1**	35.7 ± 0.5	58.3 ± 0.3	6.0 ± 0.4^*∗∗∗*^
Fullerene **2**	37.0 ± 0.8	56.6 ± 0.8^*∗*^	6.4 ± 0.2^*∗∗∗*^
48 h			
Control	46.2 ± 1.7	47.6 ± 1.3	6.2 ± 0.5
Fullerene **1**	43.1 ± 0.7	50.6 ± 0.5^*∗*^	6.3 ± 0.7
Fullerene **2**	37.9 ± 0.8^*∗∗*,§^	55.0 ± 0.3^*∗∗∗*,§§^	7.1 ± 0.5
72 h			
Control	46.7 ± 0.7	47.5 ± 0.5	5.8 ± 0.5
Fullerene **1**	46.4 ± 0.8	47.7 ± 0.6	5.9 ± 0.3
Fullerene **2**	43.2 ± 0.6^*∗*,§^	50.7 ± 0.4^*∗∗*,§§^	6.0 ± 0.3

Cells (1 × 10^6^/mL) were exposed for 24–72 hr long-lasting treatment with 10 *μ*M of fullerene **1** and fullerene **2** and then subjected to PI staining prior to performing the flow cytometric cell cycle analysis. The percentage of each phase reported was calculated by MCycle analysis software. ^*∗*^ *P* < 0.05, ^*∗∗*^ *P* < 0.01, and ^*∗∗∗*^ *P* < 0.001 versus untreated controls; ^§^ *P* < 0.05, ^§§^ *P* < 0.01 versus fullerene **1**; Student-Newman-Keuls multiple comparisons test, ANOVA.

**Table 3:** Flow cytometric analysis of the fullerene derivatives interaction with monocytes and macrophages.

	Monocytes		MDM-LPS	
	%	MFI	%	MFI
0 *μ*M	0 ± 0.01	0 ± 0.01	0.16 ± 0.04	3.1 ± 0.4

5 *μ*M fullerene **1**-FITC	3.8 ± 0.3	3.0 ± 0.05	94.1 ± 0.06	34.7 ± 6.3^*∗∗∗*^
10 *μ*M fullerene **1**-FITC	12.6 ± 1.2	3.2 ± 0.01	98.6 ± 0.19	76.4 ± 2.8^*∗∗∗*,§^

5 *μ*M fullerene **2**-FITC	0.3 ± 0.03	3.1 ± 0.04	93.50 ± 0.23	18.7 ± 0.8^*∗∗*^
10 *μ*M fullerene **2**-FITC	1.8 ± 0.19	3.4 ± 0.08	96.93 ± 0.36	31.5 ± 1.0^*∗∗∗*,§^

Cells (1 × 10^6^/mL) were exposed for 24 h to 5–10 *μ*M of fullerene derivatives: FITC tagged and subsequently recovered and run by flow cytometer. The percentage of positive cells and the mean fluorescent intensity of FL1 channel are reported. Each value is the mean ± SEM of triplicate samples. ^*∗∗*^ *P* < 0.01, ^*∗∗∗*^ *P* < 0.001 versus untreated controls; ^§^ *P* < 0.05 versus the lower concentration (5 *μ*M). Student-Newman-Keuls multiple comparisons test, ANOVA.
